# The Average Duration of Human Life in Various Countries and at Different Epochs

**Published:** 1856-01

**Authors:** 


*The Average Duration of Human Life in Various Countries and at Different Epochs.*

In France a sixth of the population die at the end of the first year; a fifth at the end of 2 years; a third at the end of 14 years; the half at the end af 42 years; the three-fourths at the end of 69 years; the four-fifths at the end of 72 years, and the five-sixths at the end of 75 years.

Before 1789, Duvillard calculated that of 100 men, 50 live to 20 years; but since 1789 there is a marked improvement, and the observations of Bienaymé, from 1823 to 1831, prove that 60, and not 50, is the true proportion. Demontferrand says that of 100 men, 7 live to 80 years, 2 to 85, 1 to 89; and that in a million of our race there are only 640 nonagenarians (from 90 to 99 years.) Matthieu stated the number at 491, and of these only 9 reached the age of 97 years, and 4 that of 99.

Centenarians, according to Duvillard and Demontferrand, exist in the proportion of 2 in every 10,000. There are, however, privileged countries. Thus, at Carlisle, Milne found 9 in the 10,000. At most one dies in Paris every year.

Benoiston de Châteauneuf having examined 15 million lives, finds that 44 in 100 live to the age of 30; 23 to 60; 15 to 70; 4½ to 80, and 44-73ds to 90.

At present the average duration of life in France appears to be 39 years 8 months. Twenty years ago, Bienaymé valued it only at 36 years, and Demontferrand represented it at 33 years 8 months. In 1817 it was only 31 years 3 months; before 1789, according to Duvillard, 28 years 9 months; and Villermé has established that in Paris during the 18th century it was 32 years; in the 17th, 26 years, and only 17 years in the 14th century.

In France but one septagenarian is found among every 33 individuals; one octogenarian in every 160, and but one nonagenarian in 1900. Of these last there are nearly 17,500. Matthieu, however, computes that in every 174 persons there is one octogenarian, and one nonagenarian in every 1740.

At Geneva the average duration of life was 18 years 5 months in the 16th century: 23 years 4 months in the 17th, and from 32 to 33 years in the 18th; from 1815 to 1826, it has risen to 38 years 10 months.

At present in France, as we have seen, the average duration of life is 39 years 8 months, that is to say, on our birth we have before us 39 years and 8 months of probable existence; at 4 years, a period when all the favorable chances are united, we have 49 years 4 months; according to Deparcieux, we have only 40 years and 3 months at 20 years of age; 34 years and 1 month at 30 years of age; 27 years 6 months at 40; 20 years 5 months at 50; 14 years and 3 months at 60; 8 years and 3 months at 70; 4 years and 8 months at 80; and 1 year and 9 months at 90.

In 1840 the average duration of life in England was 38 years; in France 36 years and a half; in Hanover, 35 years 4 months; in Schleswig-Holstein, 34 years 7 months; in Holland 34 years; in the Duchy of Baden, 32 years 9 months; at Naples, 31 years 7 months; in Prussia, 30 years 6 months; in Wurtemburg, 30 years, and 29 years in Saxony.

Demontferrand has divided into three classes the departments where life is the longest, and also those where it is briefest. In the first class, which is that of the longest livers, there are 28 departments: Calvados, Gers, Basses-Pyrénées, Cantal, Charente, Orne, Lot-et-Garonne, Lot, Maine-et-Loire, Aveyron, Gironde, Lozére, Deux-Sèvres, Manche, Tarn-et-Garonne, Doubs, Mayenne, Dordogne, Creuse, Loire-Inférieure, Eure, Vienne, Haute-Marne, Indre-et-Loire, Haute-Loire, Ariége, and Haute-Garonne. The average duration of life for example, is 44 years 7 months in the Calvados and the Lot-et-Garonne.

The second class contains 33 departments: Jura, Puy-de-Dôme, Vendée, Sarthe, Charente-Inférieure, Corse, Seine-et-Oise, Somme, Oise, Tarn, Seine-Inférieure, Corrèze, Eure-et-Loire, Côte-d'Or, Pas de-Calais, Ardennes, Aube, Marne, Drôme, Allier, Vosges, Ille-et-Vilaine, Isêre, Yonne, Var, Meurthe, Meuse, Aude, Laudes, Herault, Ain.

The third class comprehends the other 25 departments. In Finisterê and Pyrênées-Orientales, the average duration of life is only 28 years 2 months and 28 years 1 month. Females have the advantage; thus in every 100 of each sex, at 10 years there are 58 females and 53 males alive; at 20 years, 58 females and 48 males; at 50 years, 33 females and 30 men; at 60 years, 23 females and 23 males; at 70 years, 15 females and 13 males; at 80 years, 5 females and 4 males, according to Benoiston (de Châteauneuf); and although 17 boys are born for every 16 girls, the proportion is soon re-established; thus at the end of one year for every 1000 children of each sex there are 848 girls and 823 boys alive.

These observations, made with the greatest possible accuracy, are curious in the extreme, and from them the legitimate conclusion is drawn that the average duration of life in Europe, and especially in France, is increasing every year.—*Moniteur—quoted in the Gazette des Hôpitaux, July, 14th*, 1855.

